# Transformer deep learning architectures for health economic modelling of novel therapies for Alzheimer's disease

**DOI:** 10.1002/alz70860_099719

**Published:** 2025-12-23

**Authors:** Linus Jönsson

**Affiliations:** ^1^ Karolinska Institutet, Dept for Neurobiology, Care Sciences and Society, Center for Alzheimer Research, Div of neurogeriatrics, Solna, Sweden

## Abstract

**Background:**

The introduction of new disease‐modifying therapies for Alzheimer's disease (AD) necessitates robust methods for assessing their cost‐effectiveness and impact on disease progression. Accurately predicting health care resource utilization and disease trajectories is critical for informed decision‐making and policy development.

**Methods:**

We developed a deep learning model based on transformer architecture to predict health care resource utilization and disease progression in AD patients. The model was trained on longitudinal data from the Swedish Dementia Registry (SveDem) linked with additional sources, including health care resource use, biomarkers, and mortality data. Clinical events and resource use were encoded as sequential tokens, allowing the model to learn complex temporal patterns. Model performance was evaluated in terms of its ability to predict the next event token and Mini‐Mental State Examination (MMSE) scores.

**Results:**

The model demonstrated high predictive accuracy, correctly identifying the next clinical or health care resource use event in approximately 40% of cases. Furthermore, it predicted MMSE scores with a margin of ±2 points. These results underscore the model's potential for capturing intricate relationships between clinical trajectories and health care resource utilization.

**Discussion:**

This transformer‐based model represents a promising tool for simulating the effects of novel AD therapies on health care resource utilization and long‐term health outcomes. By enabling accurate predictions of disease progression and associated costs, it offers a valuable framework for evaluating the cost‐effectiveness of emerging treatments in real‐world settings.

**Conclusion:**

Our study highlights the utility of deep learning approaches in advancing personalized medicine and health economics in AD. The integration of comprehensive data sources and sophisticated modeling techniques can enhance our understanding of disease‐modifying therapies’ implications for clinical and economic outcomes.

